# Turning the tables: A university league-table based on quality not quantity

**DOI:** 10.12688/f1000research.18453.2

**Published:** 2019-07-29

**Authors:** Adrian G. Barnett, David Moher

**Affiliations:** 1 School of Public Health and Social Work & Institute of Health and Biomedical Innovation, Queensland University of Technology, Brisbane, QLD, 4059, Australia; 2 Centre for Journalology, Ottawa Hospital Research Institute, Ottawa, Ontario, ON K1H 8L6, Canada

**Keywords:** meta-research, research quality, research reporting, league tables

## Abstract

**Background: **Universities closely watch international league tables because these tables influence governments, donors and students. Achieving a high ranking in a table, or an annual rise in ranking, allows universities to promote their achievements using an externally validated measure. However, league tables predominantly reward measures of research output, such as publications and citations, and may therefore be promoting poor research practices by encouraging the “publish or perish” mentality.

**Methods: **We examined whether a league table could be created based on good research practice. We rewarded researchers who cited a reporting guideline, which help researchers report their research completely, accurately and transparently, and were created to reduce the waste of poorly described research. We used the EQUATOR guidelines, which means our tables are mostly relevant to health and medical research. We used Scopus to identify the citations.

**Results: **Our cross-sectional tables for the years 2016 and 2017 included 14,408 papers with 47,876 author affiliations. We ranked universities and included a bootstrap measure of uncertainty. We clustered universities in five similar groups in an effort to avoid over-interpreting small differences in ranks.

**Conclusions: **We believe there is merit in considering more socially responsible criteria for ranking universities, and this could encourage better research practice internationally if such tables become as valued as the current quantity-focused tables.

## Introduction

League tables are used by universities to advertise their value, recruit staff and students, and attract funding, particularly philanthropic funding. There are many international league tables including the *Times Higher Education World University Rankings*, *QS World University Ranking* and *CWTS Leiden Ranking*. There are also national league tables, such as the *Complete University guide* in the UK, and there are also national ranking systems such as the UK Research Excellence Framework, but in this study we only consider international league tables. We also focus on research, and so we do not consider league tables or criteria that focus on teaching or service. Many universities have dedicated web pages that promote their league table rankings with news stories and graphics ^1– 3^. League tables create opportunities for universities to write positive stories based on either: i) their ranking, or ii) a large rise in their ranking as the tables are updated annually. Rankings can also be stratified by country, scientific field, or the league table’s criteria (e.g. teaching or research), offering multiple opportunities for positive stories. The league tables are made by groups that are independent of universities, and therefore give an external marker of quality.

Example quotes from university web pages concerning their position in league tables are below and these demonstrate some of the ways universities use league tables for self-promotion.

- “The University’s outstanding performance in the Leiden Ranking sent a strong signal to potential partners and collaborators that top-quality, highly cited research was produced across all disciplines.” http://tinyurl.com/y94tomgr
- “Deakin has climbed 62 places to enter the world’s top 300 universities, according to the latest prestigious QS World University Rankings [...] The latest ranking places Deakin in the top 1.1 per cent of universities in the world.” https://tinyurl.com/y9xzmtpk
- “The University of Toronto is among the best universities in the world for graduate employability, a new independent study says.” https://tinyurl.com/ydxju5xu
- “These results demonstrate that the University of Toronto is a consistent producer of impactful, world-class research across a broad range of disciplines” https://tinyurl.com/yd3uz83m

The quotes were selected to illustrate how universities value league tables. They were found by selective searching and are not a representative sample.

University managers often want to maintain a high ranking or increase their ranking in international league tables, and may implement top-down policies that encourage their staff to work in ways that will achieve this. A review of the impact of university league tables in the UK found that they, “appear to be having a significant influence on institutions’ actions and decision-making” ^4^. These changes to research practices may have societal costs. For example, encouraging researchers to focus on quantity so that rankings based on publications numbers increase, may lead researchers to cut corners in order to increase their output at the expense of quality ^5^. 

League tables could potentially be used to promote positive changes in research culture if they included criteria of good research practice, which might then encourage university managers to widely promote good practice.

### Criteria used by league tables

The International Ranking Expert Group (IREG) audit university league tables and aim to strengthen public awareness and understanding of university rankings. A recent inventory by IREG found 17 international league tables ^6^, although two are based solely on web traffic and one concerns environmental sustainability. Of the remaining 14 tables, 12 use publication numbers, and 12 use citations.

Although papers and citations are commonly used, every league table uses their own method to count them. Variations include:

- Only papers or citations from selected “high quality” journals
- Only relatively highly cited papers
- Only papers cited by industry
- Citation numbers divided by the number of papers
- Paper numbers divided by the number of staff

The differences between league tables could reflect genuine differences of opinion in the best way to use the data. It could also be somewhat due to a desire by league tables to differentiate themselves and so produce novel results. It could also be because papers and citations are imperfect proxies of quality, and so there are multiple opinions on how best to refine them.

### Criticisms of league tables

A seminal paper on institutional ranking (including hospitals and schools) in 1996 by Goldstein and Spiegelhalter stated that responsible rankings, “may provide relevant information to universities, students, funders and governments” ^7^. However, they also cautioned about the need to consider data quality, uncertainty in the rankings, gaming by institutions, and unwarranted conclusions based on small changes in ranks. A report on the use of public league tables recommended that every table should have an appropriate and prominent “health warning” about their limitations ^8^.

The criteria used by university league tables have been criticised for lacking construct validity ^9^ and for experiencing implausibly large changes from year to year ^10^, some of which were due to calculation errors and methodological changes ^11^.

A report on the use of citation statistics warned that “citation data provide only a limited and incomplete view of research quality” ^12^. An analysis of misprints in citations suggested that most researchers simply copy citations without reading the actual paper ^13^, which undermines their face validity as a ranking criteria. Citations and paper numbers can be gamed ^14, 15^, and gaming by researchers can greatly alter a university’s ranking ^11^. Concerns about the misuse of simplistic metrics in research led to the Leiden Manifesto in 2015, which set out ten principles for the proper use of metrics for evaluating researchers and institutions ^16^. In 2017 the Leiden group created ten more principles for responsibly ranking universities ^17^, which included transparency and acknowledging the uncertainty in rankings.

### Good research practice

To our knowledge, only one current international league table includes a measure of best publication practices, by which we mean established methods that increase the robustness, transparency and reproducibility of research. The one example is the *Scimago Institutions Rankings* which includes the percent of Open Access papers, however this is weighted at just 2% and far higher weightings are given to publication numbers and citations. There is an international league table of potentially questionable research practice, which is the *Retraction Watch* table of individual researchers ranked by their number of retracted papers ^18^.

Examples of good research practice are:

- Including key stakeholders in forming research questions ^19^
- Publishing a protocol and ensuring that the results presented match those planned in the protocol ^20^
- Publishing results even when they are statistically negative or potentially commercially damaging ^21^
- Using reporting guidelines to write-up the results ^22^
- Sharing data and code where available ^21^

Unlike the traditional metrics, such as the number of publications, used by current league tables, these metrics are prerequisites to solving recognised problems in science. Recent evidence points to a growing reproducibility crisis in many fields of research, which is only possible to examine when sharing of data, code, materials and methods takes place.

Good research practices help reduce research waste, which can occur when researchers cut corners in order to progress in the “publish or perish” game. Avoidable research waste is an enormous problem and an estimated 85% of the current investment in health and medical research is wasted due to poor research practice, which is billions of dollars per year ^23^.

In this paper we examine one of these good research practices by examining when authors cited an EQUATOR reporting guideline ^24^. EQUATOR stands for: Enhancing the QUAlity and Transparency Of health Research, and they are a wide-ranging suite of more than 400 guidelines that cover every common research study design. There is evidence that using a reporting guideline improves the quality of the published paper ^25, 26^. Our key assumption is that citing the guideline is an indicator of good research practice. Our aim is to reward research “soundness” rather than the typical aim of rewarding “excellence”, an approach which has failed to improve research quality and has instead fueled hyper-competition by rewarding the quantity of research ^27^. An important difference from our approach compared with previous league tables, is that we reward the universities whose researchers give the citation, not the universities of researchers who receive the citation.

There are four EQUATOR centres around the world (UK, France, Canada and Australasia) with the aim of promoting the use of the guidelines worldwide. Many of the most commonly used EQUATOR guidelines have been translated into multiple languages.

There is a wide literature on rankings and university league tables including discussions of policy ^28^, design ^29^ and statistical critiques ^7^, as well as systematic reviews ^30^ and books ^31^. We do not review this literature in detail, as our primary aim was to identify whether a league table could be constructed based on good research practice.

## Methods

We use the phrase “university rankings” to be consistent with the existing league tables. However, “institutional rankings” would be more accurate because we include research institutes that may be affiliated with universities but do not graduate students, such as the “Baker Heart and Diabetes Institute”.

### Papers included

We counted papers that cited one of the EQUATOR guidelines for clinical trials ( CONSORT) ^32^, systematic reviews ( PRISMA) ^33^, and observational studies ( STROBE) ^34^. We chose these three guidelines because they cover three commonly used study designs. Each guideline was published simultaneously across multiple journals, which was done to increase their reach into multiple fields. We therefore counted citations to any of the original papers or updates to the guidelines (see Supplementary List 1) ^35^. If a paper cited multiple EQUATOR papers, then only one was counted.

To include only papers that adhered to the first item on the CONSORT and PRISMA guideline check-lists, which is to include the study design in the title, we only included papers that included the following in their title:

- For CONSORT papers: “randomised trial” OR “randomized trial” OR “RCT”
- For PRISMA papers: “systematic search” OR “systematic review” OR “systematic literature review” OR “scoping review” OR “meta-analyses” OR “meta-analysis” (including versions without hyphens)

We did not include a restriction for STROBE papers because there are many observational study designs and any list we created might exclude valid papers.

To focus on original research, we included publication types of Articles or Reviews, and excluded Editorials, Commentaries and Corrections.

We aimed to sum citations per year and we examined the two most recent complete years of data by using papers published in 2016 or 2017.

We used *Scopus* to identify citations because it is a recognised database for citations that is used by four international league tables, and because of the ease of extracting the data using the *rscopus* package in R ^36^ (Version 0.6.3). We used the *rentrez* package in R (version 1.2.1) to extract meta-data on the papers from *Pubmed* ^37^. Papers were excluded if they did not have a digital object identifier (DOI), because this was the key linking variable for extracting the affiliation data. The data extraction from *Scopus* was performed on 19 December 2018.

### Cleaning affiliations

We extracted all authors’ countries and affiliations. The affiliation data is free text and required extensive cleaning to extract a standardised set of universities. Affiliations were changed to:

- Remove departments, for example, “Mansoura University, Urology and Nephrology Center” to “Mansoura University”
- Include non-Roman letters, for example, “Universite de Montreal” to “Université de Montréal”.
- Remove locations, for example: “Massey University, Auckland” to “Massey University”. The exception was where the location was needed to differentiate the university, for example the University of Newcastle in the UK and Australia.
- Remove unnecessary prefixes, for example: “The University of Sydney” to “University of Sydney”
- Spell-out acronyms, for example: “UCL” to “University College London”
- Consolidate dual names, for example: “University of Reykjavik” to “Reykjavik University”
- Consolidate institutes associated with a university, for example: “The Ottawa Hospital” is associated with the “University of Ottawa”. We used the list of 1,802 affiliated institutions provided by the 2018 Leiden ranking ^17^.

We changed vague affiliations to missing, for example “Faculty of Health”.

We standardised affiliations to ensure that citations were consolidated into a single university rather than being split over two or more universities and hence creating a falsely low position in our league table.

A flow chart of the data collection and management is in Supplementary Figure 1 ^35^.

### Creating our league table

To create a score per university, we summed the total number of citing papers per university per year. To better divide the credit from a citation, we used an organisational-level fractional count of author affiliations per paper ^38^. So, for example, if a paper had two affiliations in the address list, one from Queensland University of Technology and one from Ottawa Hospital Research Institute, then each university would gain 0.5. A fractional count avoids the situation where universities gain a full point even when their staff member was only one of multiple authors.

We examined the amount of missing affiliation data by country to look for biases in the affiliation data that may disadvantage particular universities or geographic regions in our league table. We also included “Missing” as a separate university, in order to show the relative importance of missing data.

We accounted for uncertainty in our league table using a bootstrap procedure ^39^. We randomly resampled with replacement from all the citing papers and recalculated each university’s score and rank. We repeated this resampling 1,000 times. To summarise this uncertainty we created a bootstrap 95% confidence interval for the rank.

We examined changes over time by comparing the ranks of universities in the top 200 in 2016 and 2017. We used a Bland–Altman plot to examine how ranks changed between these two years ^40^. For comparison, we also used a Bland–Altman plot of the *THE World University Rankings* using their research criterion, which combines a reputation survey, data on research income and paper numbers ^41^.

We qualitatively self-assessed our league table against the ten principles for responsible ranking from the Leiden group ^42^.

As a comparison to our good research practice table, we created a standard league table based on counting each university’s papers for the years 2016 and 2017. We counted articles only, not books, editorials or letters. To match our good practice table which is focused on health and medical research, we only included papers in the three subject areas of Dentistry, Health Professions and Nursing. These data were from *Scopus*.

### Clustering universities into similar groups

We present our results as a table using the total score per university per year and give an integer rank to universities in each year. This implies a monotonic order, where each university performed better than the university below it. This is unlikely to be true, and to give a better impression of performance we used clustering to group universities into five clusters. We chose five as an *a priori* opinion of the number of meaningful clusters. We used a Bayesian clustering model defined as:

$$\begin{array}{l} S(i,t)\;∼\;\text{Normal}(\bar{x}\text{[}c\text{(}i,t\text{)],}\sigma^{2}\text{),}\;i=1,\cdots ,N_{t},\\ \;t=2016,2017,\\ \;\bar{x}\text{(1)}\;\text{=}\;\gamma (1),\\ \;\bar{x}(j)\;=\;\gamma (j)+\bar{x}(j-1),\;j=2,\cdots ,5,\\ \;\gamma (j)\;∼\;\mathrm{Exponential}(1),\;j=\text{1},\cdots ,5,\\ c(i,t)\;∼\;\text{Categorical(}\pi \text{),}\\ \;\pi (j)\;=\;\frac{\delta (j)}{\sum_{j=1}^{\text{5}}\delta \text{(}j\text{)}},\;j=1,\cdots ,5,\\ \;\delta (j)\;=\;\mathrm{Uniform}(1,99/4),\;j=1,\cdots ,5,\\ \;\sigma^{2}\;∼\;\mathrm{Uniform}(0.01,1000) \end{array}$$

where *S*( *i*, *t*) is our score for university *i* in year *t*. The five cluster means ( $\bar{x}$) are ordered from low to high. For each university we estimate their cluster, *c*( *i*, *t*) ∈ *c*(1, 2, 3, 4, 5), which comes from a categorical distribution with five probabilities *π*(1), . . . , *π*(5). These probabilities came from the sum of five uniform prior distributions which were formulated so that the minimum probability for each cluster was 1% ( *π ≥* 0.01). This was an attempt to avoid small clusters of just a few universities. We only applied the clustering algorithm to universities with a score of 2 or above, which removed the large number of universities with small samples sizes and low scores. We cross-tabulated the median clusters by year to show how many universities changed between 2016 and 2017.

The data extraction and analyses were made using R version 3.5.2 ^43^. The clustering model was fitted in WinBUGS (version 1.4.3) ^44^ and we visually checked the mixing of the Markov chain Monte Carlo estimates. The data and code that created the tables is available here: https://github.com/agbarnett/league.tables.

In summary, the aim of our table was to score universities using the EQUATOR guidelines, with higher scores indicative of better research practice. We also included measures of uncertainty via the bootstrap and attempted to cluster similar universities. We report our results using the STROBE guidelines ^34^.

## Results

Our tables included 14,408 papers giving a total of 47,876 author affiliations that could be counted. The average number of affiliations per paper was 3.3.

### Missing affiliations

The number and percent of missing affiliation data are shown by country in Table 1. If the country was missing then the affiliation was also likely to be missing. The most amount of missing data was in the USA. Overall the percent of missing affiliation data was small, at just 0.5% of all affiliations.

**Table 1.** Number of complete and missing affiliation data by country for the top ten countries. “Missing” is included as a nominal country, that is the affiliation and country data were both missing. Countries ordered by number missing.

Country	Complete	Missing	% missing
*Missing*	72	55	43.3
United States	8,064	39	0.5
Italy	2,644	22	0.8
United Kingdom	5,223	16	0.3
Australia	4,187	14	0.3
Brazil	1,609	12	0.7
Canada	3,817	12	0.3
Germany	1,606	12	0.7
Spain	1,306	10	0.8
China	4,098	8	0.2
*All other* *countries*	14,991	59	0.4
Total	47,617	259	0.5

### Highest ranking regions and countries

Before examining institutions, we first examine the scores by regions and countries, and the top ten regions and countries are shown in Table 2. The rank order of the top ten was the same for the regions and countries, except for the tenth ranked country, which was Denmark in 2016 and Spain in 2017. Every region and country in the top ten had a higher total score in 2017 than 2016, reflecting an increased use of the EQUATOR guidelines. The highest ranking regions and countries in the table are familiar producers of research.

**Table 2.** Total good research practice scores for the top ten regions and countries in 2016 and 2017. These results exclude “Missing” as a nominal country or region.

Rank	Region	2016	2017
1	Western Europe	2,459	2,986
2	Northern America	1,521	1,807
3	Asia (excluding Near East)	1,279	1,658
4	Oceania	593	727
5	Latin America and Caribbean	325	424
6	Near East	86	109
7	Sub-Saharan Africa	61	89
8	Eastern Europe	46	71
9	Northern Africa	35	38
10	Baltics	5	7
Rank	Country	2016	2017
1	United States	1,074	1,269
2	China	871	1,064
3	United Kingdom	719	827
4	Australia	553	668
5	Canada	440	526
6	Italy	319	358
7	Netherlands	296	349
8	Brazil	266	345
9	Germany	220	277
10	Denmark (2016) / Spain (2017)	136	190

### Highest ranking universities

The top ten universities in each year are in Table 3. We have presented the scores in this paper to one decimal place, but would use rounded integers in public tables to discourage readers over-interpreting small differences. The University of Toronto had the highest score for papers citing the EQUATOR guidelines in both years. Although the proportion of missing affiliation data in the entire data set is small (just 0.5%), “Missing” was in the top ten in both years.

**Table 3.** Top ten ranking universities in 2016 and 2017 for our good research practice table. Universities are ordered by their score in each year. The cluster column is the median cluster from the Bayesian model, with ‘5’ the highest cluster. The rank is the median rank and 95% bootstrap confidence interval in brackets. The standard rank is based on counting each university’s annual papers.

University	Score	Cluster	Good practice Rank (95% CI)	Standard rank
	**2016**			
University of Toronto	82.8	5	1 (1 to 2)	2
University of Sydney	75.8	5	2 (1 to 2)	5
*Missing*	47.3	4	4 (3 to 12)	– ^a^
King’s College London	46.5	4	4 (3 to 10)	16
Zhejiang University	42.0	4	7 (3 to 19)	176
University College London	40.7	4	8 (3 to 17)	7
Mayo Clinic	39.7	4	9 (3 to 20)	38
West China Hospital of Sichuan University	39.1	4	9 (3 to 22)	239
Erasmus University Rotterdam	38.1	4	10 (4 to 21)	92
University of Melbourne	37.6	4	11 (4 to 20)	13
	**2017**			
University of Toronto	97.4	5	1 (1 to 1)	1
University of Sydney	67.2	5	2 (2 to 4)	5
West China Hospital of Sichuan University	56.7	5	4 (2 to 10)	206
*Missing*	56.6	5	4 (2 to 10)	– ^a^
University College London	53.8	4	5 (2 to 10)	8 ^b^
King’s College London	50.3	4	7 (3 to 13)	12
Harvard University	50.1	4	7 (3 to 12)	8 ^b^
University of Ottawa	47.4	4	9 (4 to 14)	95
Monash University	47.2	4	9 (4 to 15)	25
University of Oxford	46.8	4	9 (4 to 16)	64

^a^ There was no standard rank for missing affiliations. ^b^ Tied.

The University of Toronto was ranked highest for good research practice in both years, and there was little uncertainty in this top ranking as the bootstrap confidence intervals were rank 1 to 2 in 2016 and rank 1 to 1 in 2017. The University of Sydney was ranked second in both years.

The clustering model selected only a small number of universities to be in the highest category of ‘5’, despite our attempt to avoid small clusters by formulating a minimum prior probability of 1%. Summary statistics for the five clusters are in Supplementary Table 1 ^35^.

There was relatively little movement in clusters between years for the best clusters of ‘3’ to ‘5’ ( Table 4). There was more movement over time between the lowest two clusters of ‘1’ and ‘2’. Only two universities moved by two or more clusters, which was from ‘1’ to ‘3’.

**Table 4.** Cross-tabulation of estimated clusters for universities in 2016 (rows) and 2017 (columns). The diagonal numbers in bold correspond to no change from 2016 to 2017. ‘5’ is the highest cluster with the best score.

2016	1	2	3	4	5	Total
2017						
1	**120**	80	2	0	0	202
2	48	**129**	30	0	0	207
3	0	10	**42**	9	0	61
4	0	0	2	**16**	2	20
5	0	0	0	0	**2**	2
Total	168	219	76	25	4	492

The 95% bootstrap intervals were wider for universities outside the top ten. For example, for the university ranked 100 in 2017, the 95% interval was from rank 63 to 176. The width of the interval increased by an average of 13.6 for every 10 increase in rank (95% CI 13.0 to 14.1 using linear regression; see Supplementary Figure 2 ^35^). This increase was due to the reduced sample size (number of papers) for lower ranked universities.

The universities in our top 10 had varied results using a standard ranking, with some being in the top 10 and others outside the top 100. Two Chinese universities ranked in the top ten in our good research practice ranking, but were outside the top 100 using the standard table. Erasmus University and The University of Ottawa also did much better on the good research practice ranking that the standard ranking. The Spearman’s rank correlation between the standard ranking and our good practice ranking was 0.59.

Complete tables for all universities with a score of two or above are available online: https://aushsi.shinyapps.io/equator (available until 2020). These interactive tables allow examination of the results by year, geographical region and selected countries. The top 50 universities per year are shown in Supplementary Tables 2 and 3 ^35^.

### Agreement in ranks between years

**Figure 1.**
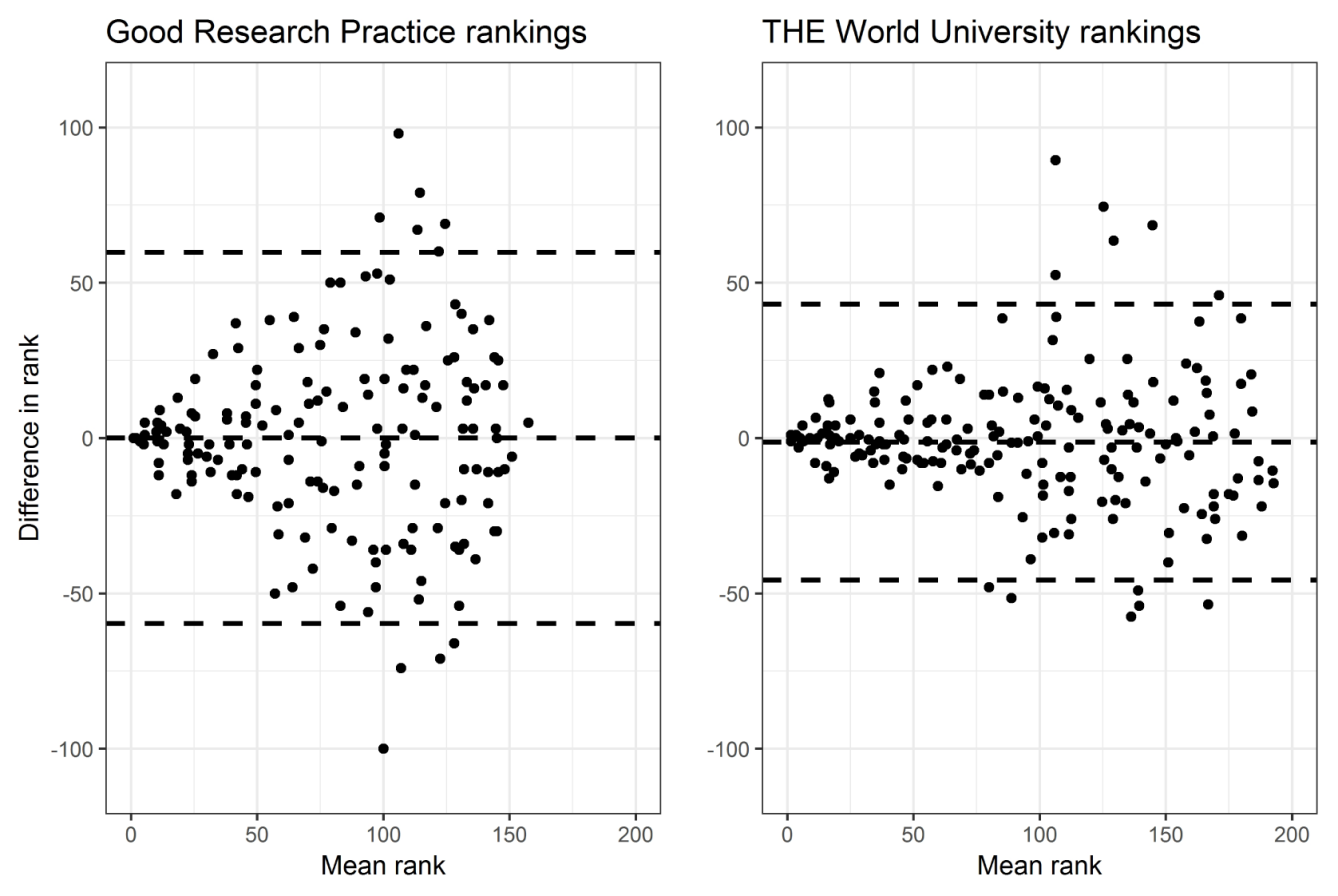
Bland–Altman plots of the agreement in university league table ranks between 2016 and 2017 for our good research practice league table and the *Times Higher Education* league table for research. We only examine universities in the top 200 in both years, which is 161 in our table and 184 in the THE table. The dashed horizontal lines are the Bland–Altman limits of agreement.

We show the agreement in university ranks between years using Bland–Altman plots in Figure 1. For both our league table and the *THE* table, there was less change in the highest ranking universities, and more movement between years at lower ranks. The Bland–Altman limits of agreement were –60 to 60 in our table and –46 to 43 for the *THE* table.

### Assessment against the ten Leiden principles for ranking universities

We assessed our Good Research Practice league table against the ten Leiden principles in Table 5.

**Table 5.** Self-assessment of our Good Research Practice league table against the ten principles for the responsible use of university rankings ^42^.

#	Principle	Self-assessment
1	A generic concept of university performance should not be used	We did not use a composite measure and detail what our score measures
2	A clear distinction should be made between size-dependent and size- independent indicators of university performance	Our score is size-dependent and we acknowledge that universities with larger health and medical research departments have more potential to achieve higher ranks
3	Universities should be defined in a consistent way	Some universities had varying affiliation wordings and we tried to appropriately combine affiliations. This was challenging and there may be combinations that we have missed.
4	University rankings should be sufficiently transparent	We have openly shared our R code that produced the tables and described our methods in this paper
5	Comparisons between universities should be made keeping in mind the differences between universities	This is a matter of how readers interpret differences between universities. To aid comparisons we could potentially add an estimate of this size of each university’s health and medical research staff.
6	Uncertainty in university rankings should be acknowledged	We used a bootstrap procedure to estimate the uncertainty in ranks.
7	An exclusive focus on the ranks of universities in a university ranking should be avoided; the values of the underlying indicators should be taken into account	We used clustering to try to more sensibly group universities by performance compared with ranks. A change in cluster between years will more likely reflect a real change compared with a change of a few league positions.
8	Dimensions of university performance not covered by university rankings should not be overlooked	We acknowledge that our table has a specific focus on health and medical research. Within this field it will be biased towards researchers producing quantitative papers, and does not currently recognise qualitative work.
9	Performance criteria relevant at the university level should not automatically be assumed to have the same relevance at the department of research group level	Our scores may be the amalgam of multiple schools in the same university, e.g., schools of public health and medicine. Care should be taken about interpreting how scores reflect the performance of individual schools or researchers (the ecological fallacy).
10	University rankings should be handled cautiously, but they should not be dismissed as being completely useless	We aimed to provide a different ranking system to current league tables, and one that might encourage good research practice.

## Discussion

Current league tables place a high value on the quantity of research outputs and citations. The irony is that the biomedical literature is littered with publications that cannot be reproduced, have substantive reporting biases and mistakes in study design, making much of such output unusable ^20^. It is hard to imagine why most universities continue to support the current ranking schemes given that they may be reducing the positive value universities have on society. We believe there is merit in considering alternative more socially responsible criteria for ranking universities.

We have created a league table based on a good research practice criterion that shows which universities are performing well and which could improve. We aimed to include all eligible universities, and so our results should be inclusive and generalisable.

### Future ranking criteria

Lindner et al recently examined whether metrics and incentives could be developed to encourage scientists to use high-quality methods and publish “negative” studies ^45^. They concluded that, “If rigorous, innovative studies of significant issues and publication of valid, reproducible results are desired, the best way to achieve those objectives is to explicitly evaluate and reward scientists based on those criteria.”

Lane suggested that new metrics should capture “the essence of what it means to be a good scientist” ^46^ and future league tables could include:

- the percent of papers that are open access (as suggested by Nichols and Twidale ^47^),
- papers where the data and/or code have been openly shared,
- studies that were pre-registered and published in a timely manner,
- papers with a published protocol.

However, league tables generally rely on large volumes of data to create scores, meaning these criteria would need to be automated. At present we could only likely automate whether matching data or protocol paper existed, and not whether the data was complete or whether the authors followed the protocol. Detailed data that cannot be automated can be collated on a smaller scale using audits ^48, 49^.

We could expand our criteria to include more of the EQUATOR guidelines, such as the STARD guidelines for diagnostic accuracy studies ^50^. Including more EQUATOR guidelines would increase the sample size per university and so would likely reduce some of the variation between years shown in Figure 1.

We did not adjust for the size of the university to produce a relative measure of performance. Hence our table is biased towards larger universities that have more staff, an issue recognised by the Leiden manifesto on metrics ^17^. An ideal standardisation would be to adjust for the number of papers that failed to cite an EQUATOR guideline when appropriate. This could be used to give an indication of performance regardless of size, and would also show the potential improvement for each university.

One surprising result from our tables was the high rank of “Missing”. This shows the importance of correctly completing affiliations, and universities could increase their rankings (in our table and others) by promoting a clear and consistent affiliation to their staff. We recommend that all league tables report the amount of missing data and show its ranking in their tables. We also recommend, as have others ^7, 17^, that all league tables include a measure of ranking uncertainty.

### Limitations

There are many limitations to constructing a university league table, and our tables should be treated as suggestive rather than definitive ^7^.

It is impossible to numerically validate our table because there is no gold standard ranking against which we can compare our results. We qualitatively assessed our own performance against the ten Leiden principles, but others may be more critical.

A valid concern with our table is that it would be gamed, with researchers simply citing an EQUATOR guideline without engaging with it. This is very likely to happen, but we cannot estimate the scale of this problem. This is less likely in journals that appropriately implement reporting guidelines because there is an internal check. The harms from such gaming could be outweighed by the number of researchers and universities that genuinely engage with the EQUATOR guidelines. Benefits would likely include greater awareness of the guidelines, and prompting researchers who were already aware of them to use them more rigorously. Complete and transparent reporting has been indicated as an essential prerequisite in dealing with the reproducibility crisis ^51^. Some token engagement with a guideline could be spotted by the paper’s peer reviewers, although peer reviewers often have limited time and have an imperfect record of spotting mistakes in papers ^52^. It may be possible to automate how the paper has adhered to the guidelines and produce a report that is shared with the authors, reviewers and editor(s), and there is an ongoing trial at the journal *BMJ Open* of such a tool ^53^.

The free text affiliation data from *Scopus* were challenging to process as they were often incomplete and inconsistent. Some universities have multiple versions of their name, including acronyms and English-language versions. We made extensive searches and asked international colleagues to check where consolidations could be made. However, we are very likely to have missed some consolidations, and hence some universities may be too low in our tables because their data has been spread across multiple names. Unfortunately we were unaware of the *Global Research Identifier Database project* https://www.grid.ac/ which helps to standardise institution names, and incorporating this data could improve our table accuracy.

We tried to examine a correlation in ranks between our tables and those of the *Times Higher Education World University Rankings* and *CWTS Leiden Ranking*. However, it was very difficult to correctly merge the data because of the large variation in affiliation names. Just one of many examples is we use “Mayo Clinic”, whereas the *Times Higher Education* uses “Mayo Medical School”, and this institute is not included in the *CWTS Leiden Ranking*.

### Related study

We could only find one previous related study, which was an international ranking that aimed to measure research quality by using membership on academic editorial boards of professional journals ^54^. They extracted researchers’ names from the websites of 115 economics journals creating a sample of over 3,700 researchers, and created league tables of researchers and universities. Their conclusion was that their table could be used to find experts to evaluate research quality.

## Conclusions

International league tables are fuelling a hyper-competitive research world that values quantity over quality. We attempted to create the first international league table that focused on good research practice. This is part of a long recognised need to focus on quality over quantity, which was raised by Doug Altman in 1994 when he said, “We need less research, better research, and research done for the right reasons” ^55^. Our table is not a perfect measure of research quality, but we hope that such tables will become valued by right-thinking universities whose goal should be to produce robust research rather than simply the most amount of research.

## Data availability

### Underlying data

A random selection of 500 rows of the data has been made available (see below). The public sharing of data for the purpose of reproducibility with a specific party is permissible upon written request and explicit written approval and the dataset remains with the customer/research. Requests can be made to: integrationsupport@elsevier.com. Zenodo: agbarnett/league.tables: Ready for journal submission. https://doi.org/10.5281/zenodo.2594016 ^35^.

### Extended data

Zenodo: agbarnett/league.tables: Ready for journal submission. https://doi.org/10.5281/zenodo.2594016 ^35^.

- Supplementary List 1. List of papers for which citations were counted.
- Supplementary Figure 1. Flow chart of the data collection and management steps.
- Supplementary Table 1. Summary statistics for the five clusters from the Bayesian model. Estimated probability for each cluster ( *π*), mean scores $\bar{x}$ , and 95% credible intervals for means.
- Supplementary Table 2. Top 50 ranked universities in 2016.
- Supplementary Table 3. Top 50 ranked universities in 2017.
- Supplementary Figure 2. Scatter plot of the width of the 95% bootstrap interval against rank using the top 200 universities in both years.

Where appropriate, extended data are held under the MIT License.

## Software availability

**Source code used for analysis available from:** https://github.com/agbarnett/league.tables.

**Archived data and code at time of publication:** https://doi.org/10.5281/zenodo.2594016 ^35^.

**Licence:** MIT License
