# Supplementary material for: Turning the tables: A university league-table based on quality not quantity
Source: F1000Res. 2019 Jul 29;8:583. Originally published 2019 Apr 29. [Version 2] doi: 10.12688/f1000research.18453.2 (PMC6611132; doi:10.12688/f1000research.18453.2)
Supplement: Peer review history

# Peer review: Reviewer response for version 2

**DOI:** 10.5256/f1000research.22006.r51753

**Published:** 2019-07-30

**Authors:** David M. Nichols

**Affiliations:** 1 Department of Computer Science, University of Waikato, Hamilton, New Zealand

No further comments.

I confirm that I have read this submission and believe that I have an appropriate level of expertise to confirm that it is of an acceptable scientific standard.

# Peer review: Reviewer response for version 1

**DOI:** 10.5256/f1000research.20190.r49068

**Published:** 2019-07-04

**Authors:** David M. Nichols

**Affiliations:** 1 Department of Computer Science, University of Waikato, Hamilton, New Zealand

This paper explores a new approach for ranking universities through a proxy for good research practice.  

 A positive aspect of the paper is that rather than simply complaining about the methods of existing ranking systems the authors have attempted to practically provide an alternative. I agree that no other ranking attempts to rank mainly (or entirely) by best practice, and this is a novel contribution of this paper. As such the paper provides a valuable counter-balance to the current dominant ranking methods. Although the paper focuses on the global rankings the same arguments would seem to apply to national ranking systems such as the REF (UK) and the PBRF (New Zealand). It would be good to add a note to this effect.

 One linkage to prior work which could be added is the discussion around research soundness in Moore *et al.* ^1^. In discussing “soundness” rather than “excellence” they say:

 “… our focus should be on thoroughness, completeness, and appropriate standards of description, evidence, and probity rather than flashy claims of superiority—presents an alternative to the existing notions of “excellence” … “Soundness” can be assessed by how it supports socially developed and documentable processes and norms.” ^1^

 These goals seem to be similar in spirit to the EQUATOR guidelines used in this paper; indeed, this paper could be regarded as an exploratory attempt to implement the “soundness” concept.

 The variability of institution names is a perennial problem in these types of study: did the authors consider standardising via the Global Research Identifier Database ( GRID)?

 This public domain data set of institutions has been produced by Digital Science as part of their Elements/Altmetrics work and is designed for this type of application.

 Scopus does have an open access indicator which is viewable in their web interface: does this value come through the API that was used? If so, it might be worth noting this data as one way to investigate alternative indicators.

 Also worth mentioning is that at least one university ranking exercise, the SCImago Institutions Rankings, does (from 2019) include a measure that rewards more Open Access publications: find here.

 “It is hard to imagine why most universities continue to support the current ranking schemes given that they may be reducing the positive value universities have on society.”

 I don’t find this hard to imagine at all, reasons could include: inertia, perceived lack of ability for an individual university to alter the rankings environment, historical autonomy of universities and consequent difficulty of coordinated global action. The institutions at the top of current rankings are probably fairly content with their position and those further down tend to have less influence/power. The paper’s criticism of universities “support” for current ranking schemes can be equally levelled at governments who organise national ranking systems: why should they not evaluate research on the grounds of good practice?

 I would include the term ‘Scopus’ in the Methods section of the Abstract as the data source is a critical aspect of scientometric studies.

 Clustering is an appropriate method to use to address the issue of small movements in ranks between years essentially being noise. The clustering definition appears fine although I don’t have experience with Bayesian Clustering so cannot give a fully informed judgement on that specific aspect. However, the key point of the paper does not depend on whether this clustering method is the best or most appropriate.  Any broadly equivalent method would be fine. I do not anticipate that the precise numbers/ranks/clusters in this paper would be actually used for ranking institutions in any consequential manner.

 The key message of the paper is that alternatives to the current ranking systems are feasible. As such it makes a useful contribution to research policy.

I confirm that I have read this submission and believe that I have an appropriate level of expertise to confirm that it is of an acceptable scientific standard.

## References

[1] : “Excellence R Us”: university research and the fetishisation of excellence. *Palgrave Communications*.2017;3(1) : 10.1057/palcomms.2016.105 10.1057/palcomms.2016.105

# Peer review: Reviewer response for version 1

**DOI:** 10.5256/f1000research.20190.r47855

**Published:** 2019-05-13

**Authors:** Ellen Hazelkorn

**Affiliations:** 1 BH Associates, and Higher Education Policy Research Unit (HEPRU), Technological University Dublin, Dublin, Ireland

This paper presents an interesting perspective on rankings with particular attention given to quality vs quantity argument. There is an extensive literature on rankings and research; the authors refer to some of the key texts. The issue of citations is arguably more problematic than referenced here; the problem of citations includes self-citation and poor practice as well as whether it is a meaningful measure of quality. However, there is a wider debate around whether citations are a meaningful or appropriate measure of 'impact' given that policy attention is increasingly on impact beyond the academic community. 

 The authors use examples of research best practice to ask about the extent to which rankings - which predominantly measure research output - are 'genuinely' concerned about the quality of the research or simply the number of papers or the number of citations, etc. This is an admirable attempt to get behind the rankings.

 As part of the methodological steps taken, the authors encounter a major default with the research data as evidenced by the way in which authors describe their own institutional/university affiliation. This is a major problem for institutions; on the other hand, it can also be one of the cheapest ways to improve in the rankings simply by cleansing the data as well as ensuring that the institutional data supplied is accurate. There are examples of universities which have changed position, up and down, because of this. Surprising how much institutional data is either inaccurate or indeed 'gamed'. The extent of the cleansing problem revealed is nonetheless staggering. 

 The results are particularly interesting, particularly the positioning of China in the country and university rankings. This comes at a time when China is being accused of poor practice, this is a very interesting result. It also reflects the increasing multi polarity of global science. While previous decades saw the EU, Japan and the US dominate, as Leydesdorff, Wagner, & Adams (2013) ^1^ argue, today the number of scientific nations now includes more than 40 nations. This is an interesting finding and may challenge perceptions of the scientific world.

 The paper gives us food for thought albeit it is unlikely to affect the main rankings - Times Higher Education and QS - or even Shanghai's Academic Ranking of World Universities given the level of complexity. Nonetheless, it asks valid questions about whether what is measured is what we think is measured.

I confirm that I have read this submission and believe that I have an appropriate level of expertise to confirm that it is of an acceptable scientific standard.

## References

[1] : International collaboration in science: the global map and the network. *El Profesional de la Informacion*.2013;22(1) : 10.3145/epi.2013.ene.12 87-95 10.3145/epi.2013.ene.12
